# Lentivirus-mediated RNAi knockdown of insulin-like growth factor-1 receptor inhibits the growth and invasion of hepatocellular carcinoma via down-regulating midkine expression

**DOI:** 10.18632/oncotarget.13027

**Published:** 2016-11-02

**Authors:** Cai Qun Bie, Xu You Liu, Ming Rong Cao, Qiu Yan Huang, Hui Jun Tang, Min Wang, Guo Li Cao, Ting Zhuang Yi, Sheng Lan Wu, Wei Jie Xu, Shao Hui Tang

**Affiliations:** ^1^ Department of Gastroenterology, The Affiliated Shenzhen Shajing Hospital, Guangzhou Medical University, Shenzhen, China; ^2^ Department of Gastroenterology, The Fifth Affiliated Hospital of Guangzhou Medical University, Guangzhou, China; ^3^ Department of General Surgery, The First Affiliated Hospital, Jinan University, Guangzhou, Guangdong, China; ^4^ Department of Gastroenterology, The First Affiliated Hospital, Jinan University, Guangzhou, China

**Keywords:** IGF-1R, HCC, lentiviral vector, RNA interference, gene therapy

## Abstract

The insulin-like growth factor-1 receptor (IGF-1R) overexpression contributes to the development of a variety of cancers. The present study explored the role of IGF-1R in the development and progression of hepatocellular carcinoma (HCC) and the possibility of IGF-1R silencing by lentivirus-mediated RNA interference (RNAi) as a therapeutic target for HCC. We showed that IGF-1R mRNA was up-regulated in Huh7 and Hep3B cells and human HCC tissues, and that IGF-1R knockdown by RNAi led to decreased proliferation, apoptosis induction, and decreased migration and invasion of Huh7 and Hep3B cells. Further, the *in vivo* study indicated that IGF-1R knockdown markedly diminished the tumorigenesis and metastasis of Huh7 xenograft. Moreover, the intratumoral administration of lentivirus-IGF-1R siRNA led to significant tumor growth inhibition in an established Huh7 xenograft model. Mechanistic investigations showed that midkine was found to be the most significantly down-regulated protein in Huh7 cells with IGF-1R knockdown, and ectopic overexpression of midkine significantly rescued inhibition of Huh7 cell proliferation, migration, and invasion caused by IGF-1R suppression. Collectively, these data suggest that IGF-1R inhibition by RNAi can significantly suppress HCC growth and invasion at least partially through down-regulating midkine expression, and IGF-1R is a potential target for HCC gene therapy.

## INTRODUCTION

Hepatocellular carcinoma (HCC) is the fifth most prevalent human cancer globally, and ranks third in the cause of cancer death [1]. It has several malignant biological properties including aggressive growth, frequent metastases, and poor survival rates, and thus it demands in-depth studies on prevention and treatment of HCC to lighten the heavy disease burden [2]. Therapy of HCC includes surgery, radiation, transarterial chemoembolization, transplantation, and systemic chemotherapy in various combinations with only marginal to moderate benefit [3–6]. However, there are no effective approaches in treating the nonresectable HCC, so novel therapeutic targets are urgently needed.

The insulin-like growth factor-1 receptor (IGF-1R), which plays an important role in regulation of embryonic development and cellular differentiation by activating downstream PI3K/Akt signal pathway, belongs to the insulin receptor family of receptor tyrosine kinases [7, 8]. Recent studies have shown that IGF-1R overexpression is linked with the raising risk of the development of various cancers including HCC [9, 10]. Furthermore, IGF-1R expression and activation is associated with resistance to cancer therapy such as chemotherapy and radiotherapy [11]. Theses researches provide new evidence that IGF-1R is an important target for cancer treatment. It has been shown that R1507, a monoclonal antibody targeting the human IGF-1R, results in the inhibition of the proliferation of several cancers, such as lung, breast, prostate cancers, and postpones the growth of xenograft tumor in nude mice [12]. However, the application of small molecule inhibitors such as RNA interference (RNAi) targeting IGF-1R for gene therapy of HCC has not yet been explored thoroughly.

RNAi is a specific gene silencing phenomenon induced by double-stranded RNA. It is one of the greatest advances in modern biotechnology, achieving the direct viewing of the loss of function of specific genes in mammalian cells, and is able to be applied to gene function studies and gene therapy for cancers and other diseases [13–15]. In this study, we constructed the lentiviral vector of short harpin RNA (shRNA) against IGF-1R gene to explore its role in the development and progression of HCC and to assess the feasibility of IGF-1R knockdown by RNAi for HCC treatment *in vitro* and *in vivo*.

## RESULTS

### IGF-1R mRNA is up-regulated in HCC cells and human HCC tissues

**Figure 1:**
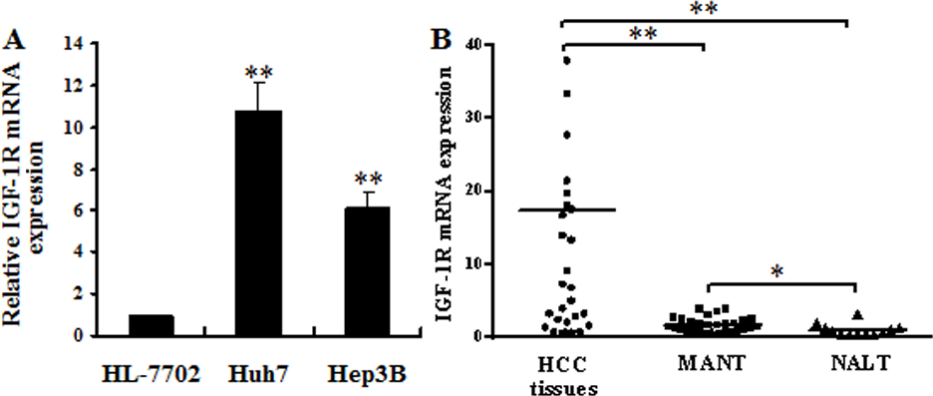
IGF-1R mRNA overexpression in HCC cells and human HCC tissues (**A**) IGF-1R mRNA expression was detected in HCC cells. The IGF-1R mRNA level was significantly up-regulated in Huh7 and Hep3B cells compared with HL-7702 cells. **P < 0.01 versus HL-7702 cells. (**B)** IGF-1R mRNA expression was detected in 29 human HCC tissues, 29 MANT, and 12 NALT. The IGF-1R mRNA level was significantly up-regulated in HCC tissues compared with MANT and NALT. *P < 0.05 versus NALT, **P < 0.01 versus MANT or NALT. HCC, hepatocellular carcinoma; MANT, matched adjacent nontumorous tissues; NALT, normal adult liver tissues.

To investigate the role of IGF-1R in HCC, we examined the level of IGF-1R mRNA in HCC cells, and in 29 human HCC tissues, 29 matched adjacent nontumorous tissues (MANT), and 12 normal adult liver tissues (NALT) (obtained from the patients with hepatic hemangioma and hepatic rupture) by quantitative RT-PCR. The result revealed that IGF-1R mRNA was significantly up-regulated in Huh7 and Hep3B cells (Figure 1A) and the HCC tissues (Figure 1B) compared with HL-7702 cells (a human normal liver cell line), and MANT and NALT, respectively, and that the expression of IGF-1R mRNA was also higher in MANT than that in NALT (Figure 1B). The mean of the relative IGF-1R mRNA level was 17.38 for all the HCC tissues studied. We arbitrarily considered samples with the relative IGF-1R expression equal to or above 17.38 as high IGF-1R expressers and samples with the relative IGF-1R expression less than 17.38 were considered as low expressers. The HCC patients with high IGF-1R mRNA expression were more often associated with poor tumour differentiation (Edmondson grade III–IV) and tumour embolus of portal vein (TEPV) compared with the patients with low IGF-1R mRNA expression, whereas there were no differences between the high and low IGF-1R mRNA expression groups regarding age, sex, tumor size, and AFP level (Table 1). The above results suggest that IGF-1R overexpression is involved in HCC development and is closely correlated to poor tumour differentiation and TEPV in patients with HCC.

**Table 1:** Comparisons of clinicopathological data in HCC patients with high and low IGF-1R mRNA expression

Parameters	IGF-1R mRNA (high, *n* = 10)	IGF-1R mRNA (low, *n* = 19)	*P*
Mean age (years)	50.50 ± 12.76	54.47 ± 13.90	0.459
No. of female patients	2	6	0.675
Mean tumor diameter (cm)	7.75 ± 3.99	7.09 ± 2.52	0.642
No. of patients with AFP ≥ 400 μg/L	6	9	0.700
No. of patients with Edmondson grade III–IV	8	6	0.021
No. of patients with TEPV	9	6	0.005

Abbreviations: TEPV, tumor embolus of portal vein; AFP, α-Fetoprotein.

### Lentivirus-mediated RNAi efficiently suppresses the expression of IGF-1R mRNA and protein in HCC cells

**Figure 2:**
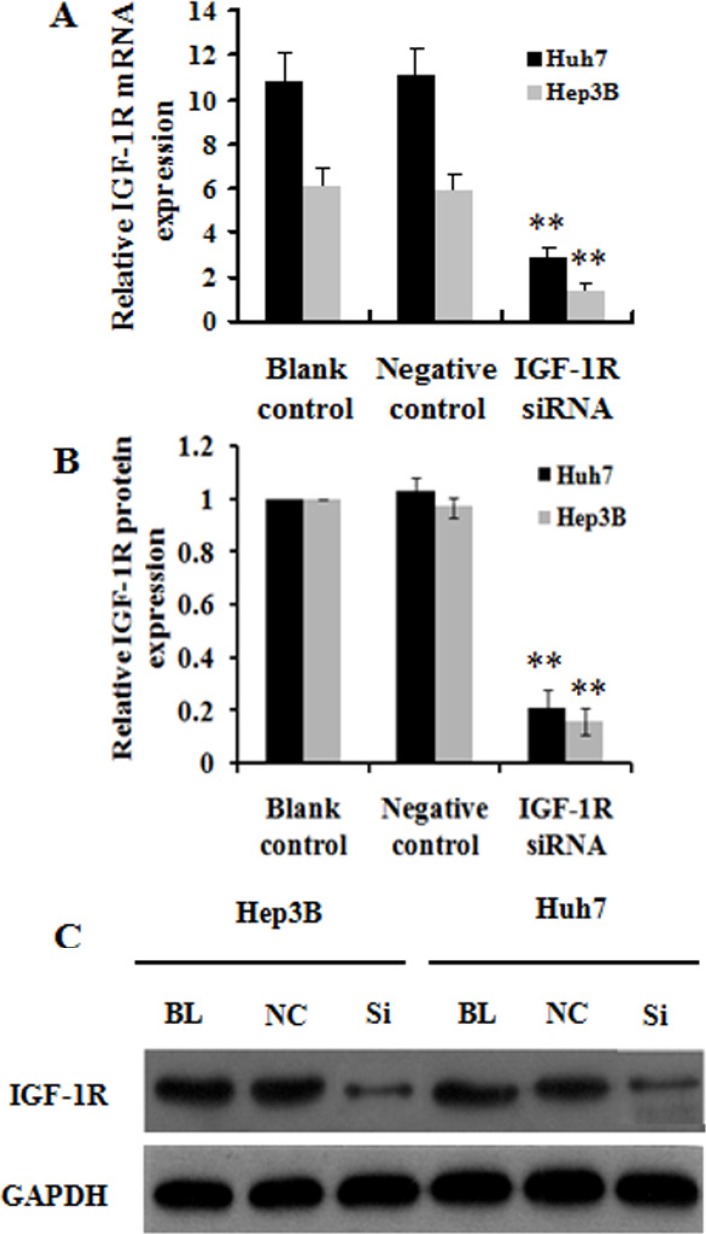
Effect of IGF-1R knockdown by RNAi on IGF-1R expression in Huh7 and Hep3B cells (**A**) IGF-1R mRNA level was significantly reduced in Huh7 and Hep3B cells with IGF-1R knockdown compared with the negative control or blank control. (**B**) IGF-1R protein level was lower in Huh7 and Hep3B cells with IGF-1R knockdown than that in the negative control or blank control group. (**C**) Representative western blot results of IGF-1R protein expression in Huh7 and Hep3B cells with IGF-1R knockdown and in the negative control or blank control group. **P < 0.01 versus negative control or blank control group. BL, blank control; NC, negative control; Si, IGF-1R siRNA.

We constructed lentiviral shRNA vector targeting the IGF-1R gene, and produced the lentiviral particles (lentivirus-IGF-1R siRNA and lentivirus-negative control). Huh7 and Hep3B cells were infected with the above lentiviruses, and the positive transductants (the cell lines stably expressing IGF-1R siRNA and negative control) were obtained. Quantitative RT-PCR showed that the level of IGF-1R mRNA in Huh7 and Hep3B cells stably expressing IGF-1R siRNA was reduced by about 73% and 76%, respectively, compared with the negative control or blank control (the untreated cells) (Figure 2A). In addition, western blot showed that the amount of IGF-1R protein in the cells of IGF-1R siRNA group was also decreased greatly compared with the negative control or blank control group (Figure 2B and 2C). These results indicate that the lentivirus-mediated IGF-1R silencing can efficiently down-regulate IGF-1R expression in Huh7 and Hep3B cells.

### Knockdown of IGF-1R significantly inhibits the proliferation of HCC cells

**Figure 3:**
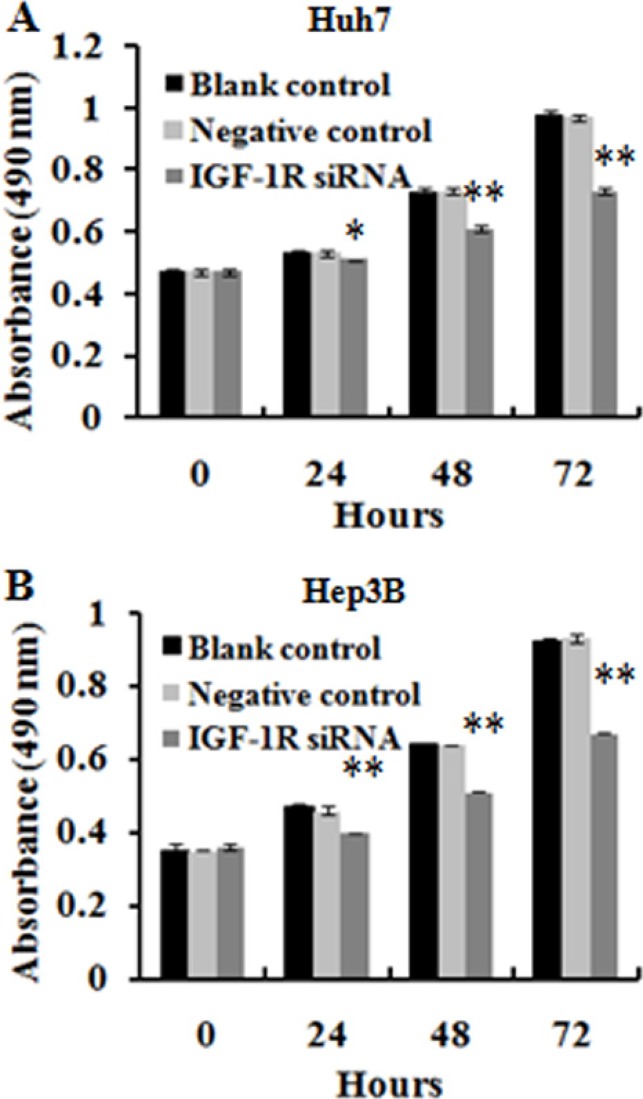
Effect of IGF-1R knockdown by RNAi on the proliferation of Huh7 and Hep3B cells Cell proliferation was evaluated by MTT, and the results of cell growth were expressed by absorbance at 490 nm. (**A** and **B**) The proliferation inhibition of Huh7 and Hep3B cells with IGF-1R knockdown was observed compared with negative control and blank control at 24 h, 48 h, and 72 h. *P < 0.05, **P < 0.01 versus negative control or blank control group.

To determine the effect of RNAi-mediated IGF-1R silencing on the proliferation of HCC cells, we carried out a MTT assay. The result showed that treatment of both Huh7 and Hep3B cells with lentivirus-mediated IGF-1R RNAi was associated with a time-dependent (from 24 h to 72 h after transfection) inhibition of the cell proliferation, whereas no significant inhibitory effect was observed in the cells treated with negative control, or in the untreated cells (blank control) (Figure 3A and 3B). This finding suggests that IGF-1R plays an important role in HCC cell proliferation.

### Knockdown of IGF-1R induces the apoptosis of HCC cells

**Figure 4:**
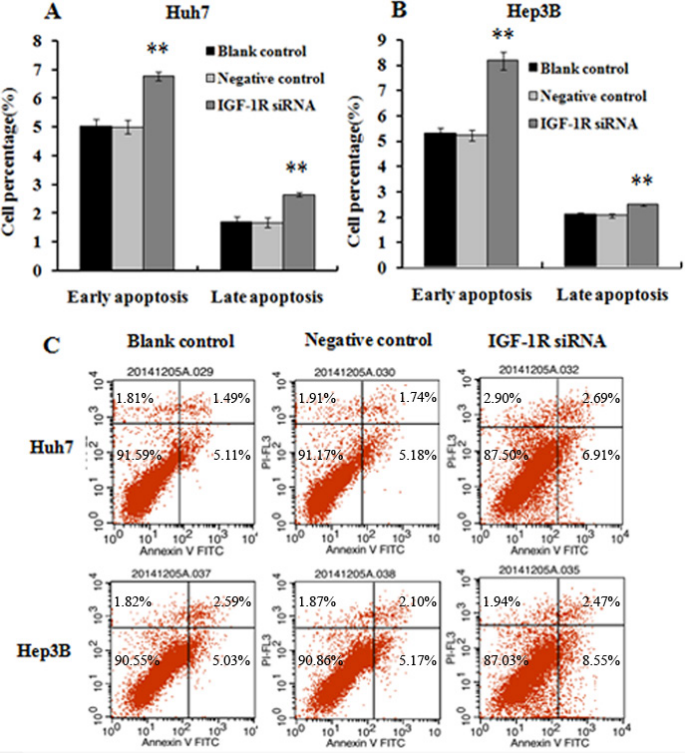
Effect of IGF-1R knockdown by RNAi on the apoptosis of Huh7 and Hep3B cells (**A**, **B** and **C**) The percentage of early and late apoptotic cells was higher in Huh7 and Hep3B cells with IGF-1R knockdown than that in negative control or blank control cells. **P < 0.01 versus negative control or blank control group.

The effect of knockdown of IGF-1R expression by RNAi on HCC cell apoptosis was investigated by flow cytometry. Silencing of IGF-1R significantly increased the percentage of early and late apoptotic cells in both Huh7 (Figure 4A and 4C) and Hep3B (Figure 4B and 4C) cells compared with the cells treated with negative control, or the untreated cells (blank control). This outcome indicates that IGF-1R inhibition promotes the apoptosis of the HCC cells.

### Knockdown of IGF-1R inhibits the migration and invasion of HCC cells

**Figure 5:**
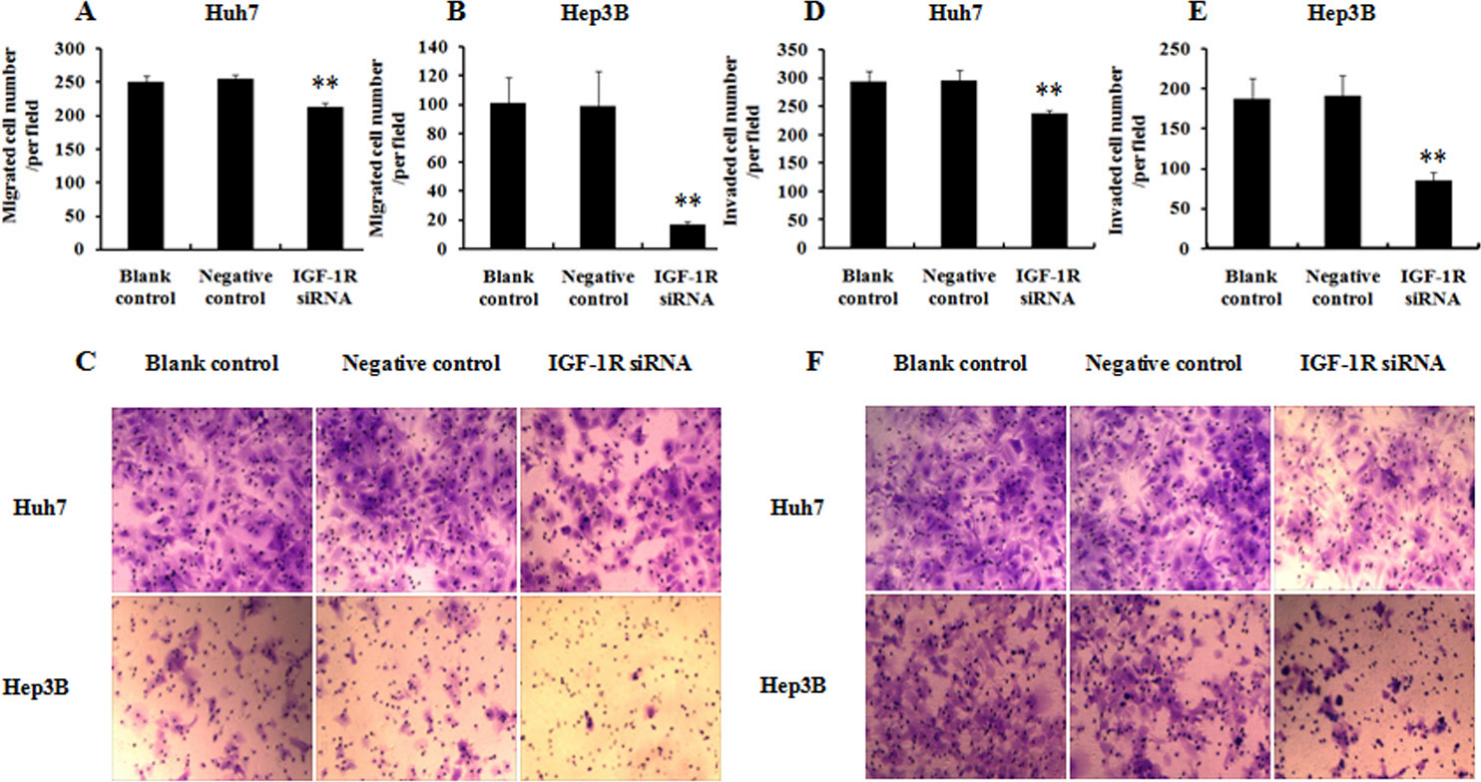
Effect of IGF-1R knockdown by RNAi on the migration and invasion of Huh7 and Hep3B cells (**A**, **B** and **C**) Analysis of migration capability of HCC cells. (**D**, **E** and **F**) Analysis of invasion capability of HCC cells. Both the migrated and invaded number of Huh7 and Hep3B cells with IGF-1R knockdown was significantly decreased compared with negative control or blank control. **P < 0.01 versus negative control or blank control group.

To evaluate whether knockdown of IGF-1R expression by RNAi was able to affect the migration and invasion capability of HCC cells, the transwell assays were performed. The results indicated that knockdown of IGF-1R expression remarkably decreased the migrated (Figure 5A–5C) and invaded (Figure 5D −5F) number of both Huh7and Hep3B cells compared with the cells treated with negative control, or the untreated cells (blank control). These results indicate that knockdown of IGF-1R inhibits the migration and invasion capability of the HCC cells.

### Knockdown of IGF-1R decreased AFP secretion of HCC cells

**Figure 6:**
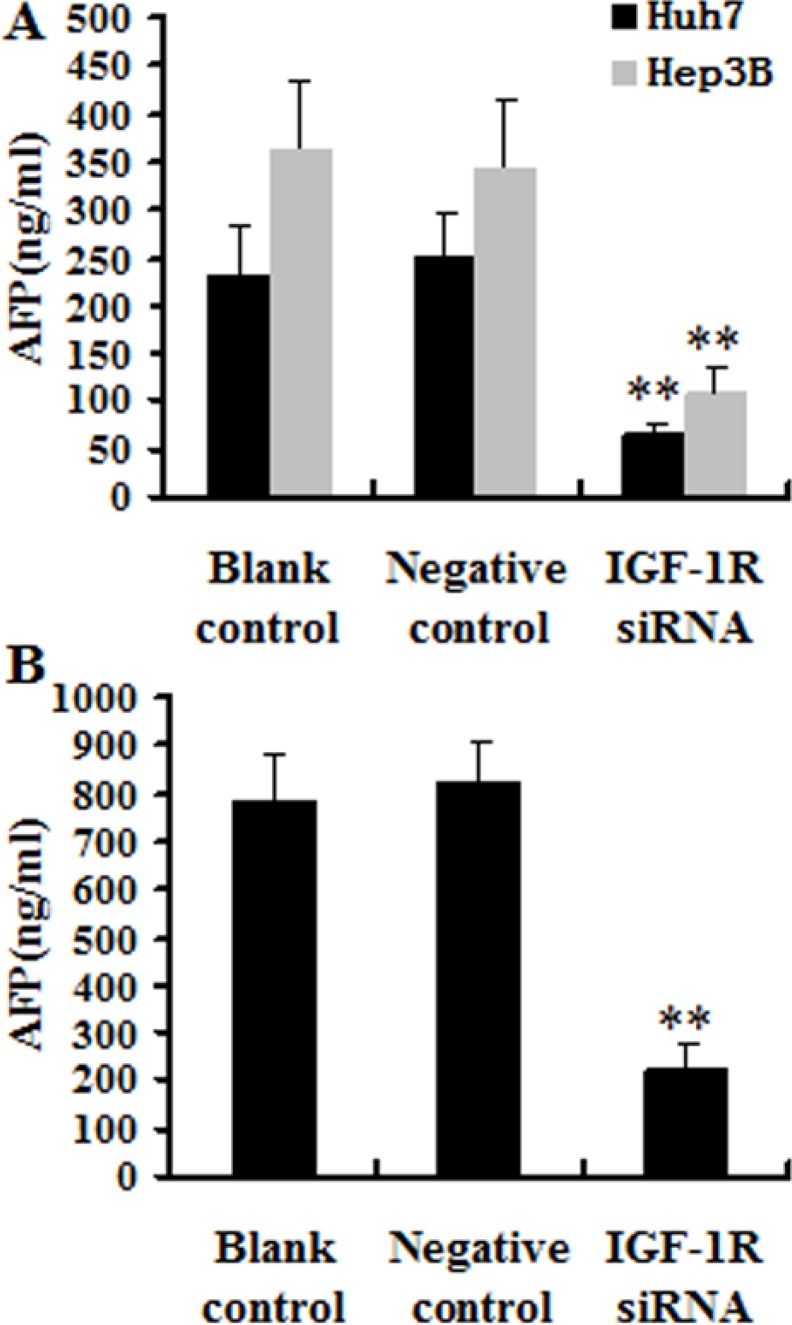
Effect of IGF-1R knockdown by RNAi on the AFP secretion (**A**) Huh7 and Hep3B cells were cultured, and two days later, the culture supernatants were analyzed. The level of AFP secretion of Huh7 and Hep3B cells with IGF-1R knockdown was significantly decreased compared with negative control or blank control. (**B**) The serum AFP level in the IGF-1R siRNA group mice was significantly lower compared with negative control or blank control. **P < 0.01 versus negative control or blank control group.

To value the effect of knockdown of IGF-1R expression by RNAi on cell secretion, the level of *AFP* secretion was measured in the cell culture supernatants using an chemiluminescent immunoassay (CLIA) (Liaison^®^, Diasorin, Torino, Italia). The results indicated that silencing of IGF-1R significantly decreased the AFP level of both Huh7 and Hep3B cells compared with the cells treated with negative control, or the untreated cells (blank control) (Figure 6A).

### Knockdown of IGF-1R suppresses Huh7 xenograft tumorigenesis and metastasis in nude mice

**Figure 7:**
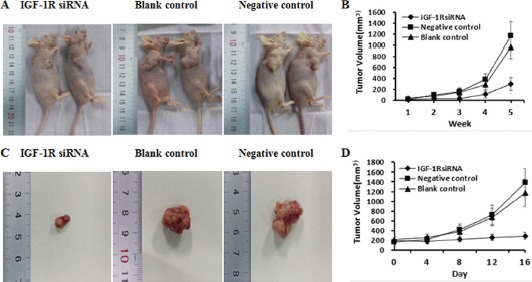
The antitumor effect of IGF-1R silencing on HCC in nude mice models (**A** and **B**) Photographs of nude mice bearing xenograft tumors and tumor volume curve of tumor growth. There were significant differences between IGF-1R siRNA and negative control or blank control group (*n* = 8/group) (*P* < 0.05) for each week from week 4 to week 5. (**C** and **D**) Photographs of tumors isolated from the nude mice 16 days after inoculation and tumor volume curve of tumor growth. There were significant differences between IGF-1R siRNA and negative control or blank control group (*n* = 4/group) (*P* < 0.05) for every four days from day 12 to day 16.

To further evaluate the effect of IGF-1R silencing on *in vivo* tumorigenicity, Huh7 cells stably expressing IGF-1R siRNA or negative control, or untreated Huh7 cells (blank control) were inoculated subcutaneously into nude mice (*n* = 8/group). The tumors in the mice inoculated with Huh7 cells stably expressing IGF-1R siRNA grew less rapidly compared with the negative control group or blank control group mice (Figure 7A). Measurement of tumor volume showed that Huh7 cells stably expressing IGF-1R siRNA formed significantly smaller tumors than Huh7 cells stably expressing negative control, or untreated Huh7 cells (blank control) (Figure 7B). In addition, one mouse in the negative control group and two mice in the blank control group showed tumor metastasis in the lung, while no evidence of metastasis was observed in the IGF-1R siRNA group mice. Additionally, the level of the serum AFP in the IGF-1R siRNA group mice was significantly lower than that in the negative control group mice or in the blank control group mice (Figure 6B). These results suggest that knockdown of IGF-1R may inhibit Huh7 xenograft tumorigenesis and metastasis *in vivo*.

### Therapeutic effect of knockdown of IGF-1R on tumor-bearing nude mice

To assess the *in vivo* antitumor activity of lentivirus-IGF-1R siRNA as a therapeutic agent, Huh7 xenograft model in nude mice was established. The tumor volume in the tumor-bearing mice inoculated intratumorally with lentivirus-IGF-1R siRNA was markedly smaller compared with the negative control group or blank control group mice (*n* = 4/group) (Figure 7C and 7D). These findings indicate that knockdown of IGF-1R by lentivirus-IGF-1R siRNA induces a forceful antineoplastic activity in HCC *in vivo*.

### Inhibition of IGF-1R suppresses HCC growth and invasion via down-regulating midkine expression

**Figure 8:**
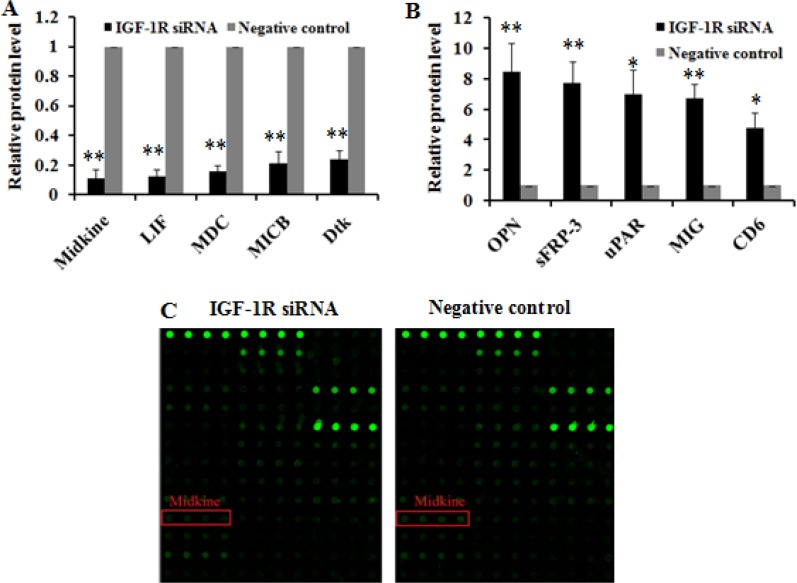
The top 10 cytokines differentially expressed due to IGF-1R knockdown by RNAi in Huh7 cells The top 5 cytokines that were down-regulated (**A**) or up-regulated (**B**) in Huh7 cells with IGF-1R knockdown were presented compared with negative control. (**C**) Imaging of midkine protein array analysis in Huh7 cells. The scanning signal was weaker in Huh7 cells with IGF-1R knockdown compared with negative control (*P* = 0.000). *P < 0.05, **P < 0.01 versus negative control group.

**Figure 9:**
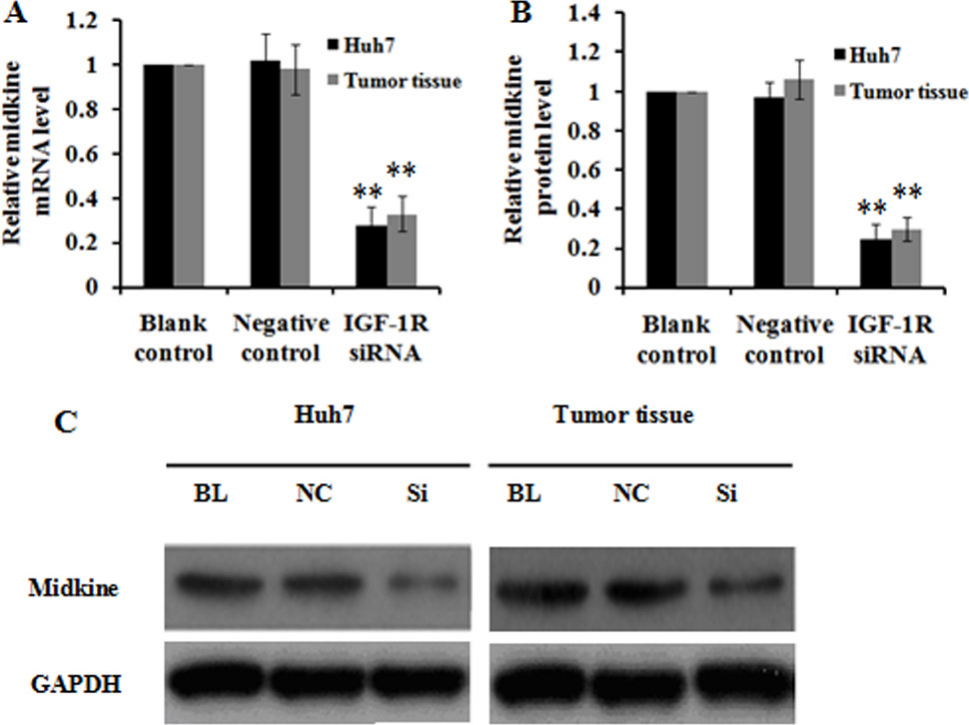
Effect of IGF-1R knockdown by RNAi on midkine expression in Huh7 cells and tumor tissues of nude mice (**A**) Midkine mRNA level was significantly reduced in Huh7 cells and tumor tissues of nude mice with IGF-1R knockdown compared with the negative control or blank control. (**B**) Midkine protein level was lower in Huh7 cells and tumor tissues of nude mice with IGF-1R knockdown than that in the negative control or blank control group. (**C**) Representative western blot results of midkine protein expression in Huh7 cells and tumor tissues of nude mice with IGF-1R knockdown and in the negative control or blank control group. BL, blank control; NC, negative control; Si, IGF-1R siRNA. **P < 0.01 versus negative control or blank control group.

**Figure 10:**
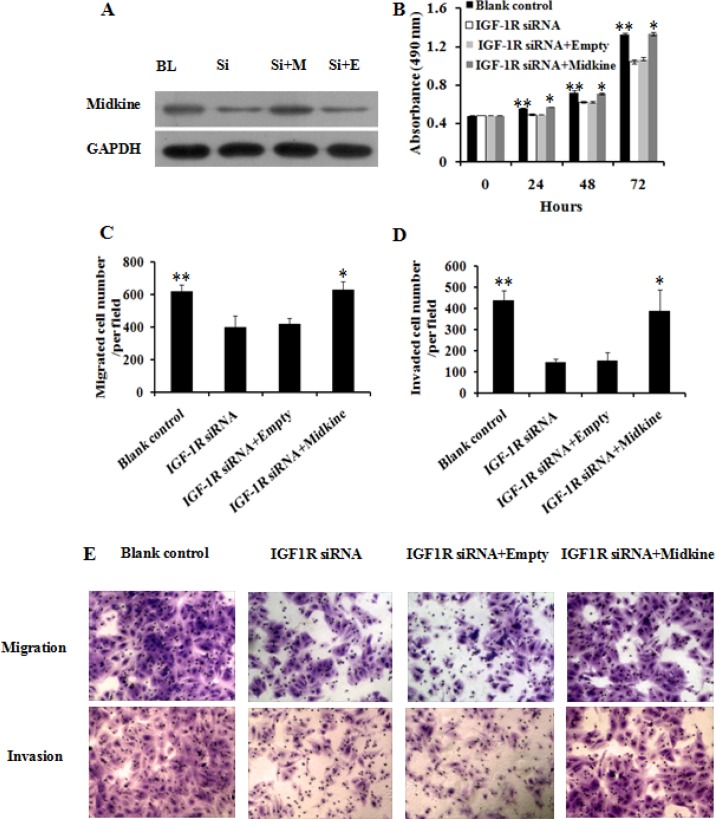
Ectopic overexpression of midkine rescued inhibition of Huh7 cell proliferation, migration, and invasion by IGF-1R knockdown Huh7 cells with IGF-1R knockdown were transfected with 1 μg midkine expression vector pcDNA3.1(+)-midkine (IGF-1R siRNA+ Midkine) or empty vector pcDNA3.1(+) only (IGF-1R siRNA+Empty). (**A**) The protein expression of pcDNA3.1(+)-midkine vector was confirm by western blot. Ectopic overexpression of midkine rescued inhibition of Huh7 cell proliferation (**B**), migration (**C** and **E**), and invasion (**D** and **E**) by IGF-1R knockdown. BL, blank control; Si, IGF-1R siRNA; Si+M, IGF-1R siRNA+Midkine; Si+E, IGF-1R siRNA+Empty. **P < 0.01 versus IGF-1R siRNA or IGF-1R siRNA+Empty group; *P > 0.05 versus blank control group.

To investigate the potential mechanism underlying inhibition of HCC growth and invasion caused by IGF-1R suppression, we employed Quantibody^®^ Human Cytokine Antibody Array 440 to identify proteins involved in IGF-1R regulation in Huh7 cells stably expressing IGF-1R siRNA. As a negative control, protein profile in Huh7 cells expressing negative control was examined in parallel. The cytokine proteins of two-fold change between Huh7 cells with IGF-1R inhibition and negative control were statistically analyzed. We found that, of the 440 human cytokines tested, which are broadly defined as secreted cell–cell signaling proteins and involved in innate immunity, apoptosis, angiogenesis, cell growth, and differentiation, a total of 79 differentially expressed cytokine proteins exhibited a more than or equal to 2-fold change. Of these, 35 were down-regulated and 44 were up-regulated. The top 5 cytokines that were down-regulated or up-regulated were shown in Figure 8A–8C. Among the above 10 deregulated cytokines, midkine, a 13-kDa small heparin-binding growth factor, has been shown to be involved in the development of multiple cancers [16, 17]. Thus, midkine was selected for the further study. We first analyzed midkine expression at both mRNA and protein level *in vitro* and *in vivo*, and the results showed that reduced midkine expression was observed in Huh7 cells with IGF-1R inhibition and in tumor tissues of nude mice inoculated intratumorally with lentivirus-IGF-1R siRNA compared with their negative controls or blank controls (Figure 9A–9C). We then explored the role of midkine in inhibition of HCC cell proliferation, migration, and invasion mediated by IGF-1R knockdown. Our results revealed that ectopic overexpression of midkine by transfecting midkine expression vector pcDNA3.1(+)-midkine into Huh7 cells with IGF-1R inhibition markedly rescued inhibition of HCC cell proliferation, migration, and invasion (Figure 10A–10E). In conclusion, these data imply that inhibition of IGF-1R suppresses HCC growth and invasion via down-regulating midkine expression.

## DISCUSSION

The insulin-like growth factor (IGF) axis includes two ligands (IGF-1, IGF-2), three cell surface receptors (IGF-1R, IGF-2R, and insulin receptor) and at least six binding proteins [18]. IGF-IR is able to influence many cellular processes including cell cycle, proliferation, migration, metabolism, and survival by mediating both IGF-I and IGF-II signaling [19]. Elevated expression level of IGF-1R has been detected in multiple human cancers, including HCC [20–23]. Suppression of IGF-1R activity is shown to induce growth inhibition, apoptosis, and cell cycle arrest in HCC [24–26]. Hence, IGF-1R has been proposed as a promising target for cancer therapeutics [27]. Several strategies of IGF-1R inhibition, such as using monoclonalantibody, antisense oligonucleotides, and small molecule kinase inhibitors directed against IGF-1R, have been developed for cancer therapy and have shown antitumor efficacy [28–30], but their clinical responses remain to be seen.

RNAi is a relatively new technology and shows promise for the development of therapeutic gene knockdown. Lentiviral vectors permit efficient delivery and stable transfection of a sequence of interest, and show minimal immunogenicity [31] and no adverse events [32]. Additionally, lentivirus can efficiently infect both dividing and non-dividing cells [33]. Thus, the combination of lentivirus and RNAi is a potential therapeutic strategy for cancer gene therapy.

In the current study, we first showed that IGF-1R mRNA was significantly up-regulated in Huh7 and Hep3B cells and the HCC tissues compared with the human normal liver cell line-HL-7702 cells, and MANT and NALT, respectively. Clinicopathological data showed that high IGF-1R mRNA expression was more frequent in HCC patients with poor tumour differentiation and with TEPV. These findings suggest the possibility that IGF-1R overexpression may play a significant role in the development of HCC and may be linked with more malignant tumour behavior.

Next, we designed the shRNA targeting human IGF-1R gene and successfully transfected it into Huh7 and Hep3B cells by lentivirus. We found that the lentivirus-mediated shRNA targeting human IGF-1R gene was highly capable of stable knockdown of IGF-1R mRNA and protein expression in Huh7 and Hep3B cells (Figure 2A–2C).

Then, we employed *in vitro* and *in vivo* experiments to further explore the role of IGF-1R in the development and progression of HCC. *In vitro* function study showed that knockdown of IGF-1R expression led to decreased proliferation, apoptosis induction, and decreased migration and invasion capability of Huh7 and Hep3B cells. Similarly, Wang et al. demonstrated that lentivirus-mediated RNAi targeting IGF-1R could led to the remarkable proliferation inhibition, apoptosis induction, and reduction of invasion activity in osteosarcoma cells [35]. The animal experiment indicated that knockdown of IGF-1R significantly suppressed Huh7 xenograft tumorigenesis, and could reduce metastatic capability of HCC xenograft in nude mice. Furthermore, the intratumoral administration of lentivirus-IGF-1R siRNA led to significant tumor growth inhibition in an established Huh7 xenograft model. Additionally, silencing of IGF-1R significantly decreased the AFP level in the culture supernatants of both Huh7 and Hep3B cells and in the serum of the nude mice. Clinically, AFP elevation is linked to more aggressive properties of HCC, such as vascular invasion, metastasis [36, 37]. As far as we know, this is the first study that demonstrates the therapeutic value of IGF-1R inhibition by lentivirus-mediated RNAi *in vivo* for HCC treatment. Taken together, these *in vitro* and *in vivo* findings suggest that IGF-1R may play a significant role in the development, progression, and metastasis of HCC, and that IGF-1R inhibition may possess the therapeutic value for HCC. Similar to our results, Tovar et al. showed that A12, a monoclonal antibody against IGF-1R, delayed tumor growth and improved survival in an HCC xenograft model [23]; Lin et al. indicated that suppressing IGF-IR expression by antisense oligonucleotides resulted in significant inhibition of tumor growth, relapse, and metastasis of orthotopic implantation model of human HCC in nude mice [25].

Finally, we explored the possible mechanism involved in inhibition of HCC growth and invasion induced by IGF-1R suppression. We conducted a protein array analysis to determine proteins related to IGF-1R inhibition, and found that midkine was the most remarkably down-regulated protein in Huh7 cells with IGF-1R silencing, which was further confirmed through quantitative RT-PCR and western blot (Figure 9). To explore the role of midkine in suppression of HCC growth and invasion by IGF-1R inhibition, we transfected midkine expression vector pcDNA3.1(+)-midkine into Huh7 cells with IGF-1R inhibition. We demonstrated that ectopic overexpression of midkine remarkably remedied inhibition of Huh7 cell proliferation, migration, and invasion caused by IGF-1R suppression, suggesting that the reduced midkine expression may contribute, at least in part, to significantly reduced HCC proliferation, migration, and invasion capability induced by IGF-1R suppression. It has been shown that midkine overexpression is observed in a variety of human cancers such as gastrointestinal cancer [17], colorectal cancer [38], lung cancer [39], breast cancer [40], pancreatic cancer [41], and HCC [42, 43]. Concordant with our findings, Rawnaq et al. indicated that the expression of midkine promoted the proliferation and migration in pancreatic cancer cells, and siRNA-mediated knockdown of midkine was associated with decreased proliferation and migration in pancreatic cancer cells [41].

In summary, the data presented in this study suggest that IGF-1R inhibition induced by lentivirus-mediated RNAi can significantly suppress HCC growth and invasion at least in part through down-regulating midkine expression, and IGF-1R is a promising target for HCC gene therapy. Our findings provide useful information for understanding the role of IGF-1R in the development and progression of HCC, and the potential mechanism involved in inhibition of HCC growth and invasion induced by IGF-1R suppression.

## MATERIALS AND METHODS

### Cell culture

Huh7, Hep3B, HL-7702, and 293T cell lines, which were purchased from American Type Culture Collection (Manassas, VA, USA), were cultured in Dulbecco's modified Eagle medium (Gibco BRL, Rockville, MD, USA) supplemented with heat-inactivated 10% fetal bovine serum (Gibco BRL). All cell lines were maintained in a 37°C incubator with 5% CO_2_.

### Construction of lentiviral shRNA vector targeting human IGF-1R gene

The small interfering RNA (siRNA) (5′-CUG ACU ACA GGG AUC UCA U-3′) targeting human IGF-1R gene (GenBank accession NM_000875) was selected for constructing the lentiviral shRNA vector. The sequences of shRNA targeting human IGF-1R gene were as follows: 5′-gatcc CTG ACT ACA GGG ATC TCA T tcaagag A TGA GAT CCC TGT AGT CAG tttttttg-3′(BamHI restriction site), and 5′-aattc aaaaaaa CTG ACT ACA GGG ATC TCA T ctcttga A TGA GAT CCC TGT AGT CAG g-3′(EcoRI restriction site). The above target sequences were synthesized and cloned into a lentiviral vector pLVX-shRNA2 (Clontech, CA, USA), which was named IGF-1R siRNA. A universal sequence that had no significant homology to any known human mRNA in the databases was cloned into the same vector and used as the negative control.

### Lentivirus production and infection

The lentiviral particles (lentivirus-IGF-1R siRNA and lentivirus-negative control) were produced by co-transfection of the above recombinant pLVX-shRNA2 vectors with Lenti-X™ HTX Packaging System (Clontech) into 293T cells according to the manufacturer's instructions. About 48 h after transfection, the lentiviruses were harvested. For cell infection, Huh7 and Hep3B cells were seeded at a density of 2 × 10^5^ in 24-well plates and transduced with the lentiviral particles. Huh7 and Hep3B cells were divided into 3 groups: the IGF-1R siRNA group (Huh7 and Hep3B cells infected with lentivirus-IGF-1R siRNA), the negative control group (Huh7 and Hep3B cells infected with lentivirus-negative control), and the blank control group (Huh7 and Hep3B cells infected without the lentiviral particles). The positive transductants (the cell lines stably expressing IGF-1R siRNA and negative control) were sorted by flow cytometry with standard FITC filter sets (ZsGreen1 in pLVX-shRNA2 vector has an excitation maximum of 493 nm and an emission maximum of 505 nm).

### Quantitative RT-PCR

Total RNA, which was extracted from the cell lines, human HCC tissue specimens, and tumor tissues isolated from the nude mice using TRIzol reagent (Invitrogen, Carlsbad, CA, USA), was reversely transcribed to obtain cDNA using 10 units of Reverse Transcriptase XL (AMV) (TaKaRa, Kyoto, Japan) according to the manufacturer's instructions. Real-time quantitative RT-PCR was performed using Power SYBR Green PCR Master Mix (Applied Biosystems, Foster City, CA, USA). The data were analyzed as described previously [44]. The primer used for the PCR was as follows: IGF-1R mRNA sense 5′-TTT CCC TTT GGA GTG TAG CT-3′and antisense 5′- CAT TGG CTG TGC AGT CAA G -3′(180bp). Each experiment was repeated thrice and all reactions were carried out in triplicate.

### Western blot

Protein extraction from the cell lines or tumor tissues isolated from the nude mice was performed as described previously [45]. Equal amounts of protein were subjected to SDS-PAGE followed by transfer of protein to nitrocellulose membranes (Bio-Rad, Hercules, CA, USA). The membranes were first incubated with the primary antibodies against IGF-1R (Abcam, Cambridge, UK) and GAPDH (Abcam), then followed by incubation with the HRP-conjugated secondary antibodies. The results were visualized using an enhanced chemiluminescence detection system (Amersham Biosciences, Piscataway, NJ, USA). Each experiment was repeated thrice and all reactions were carried out in triplicate

### Cell proliferation assay

For cell proliferation, the cells were seeded into 96-well culture plates at a density of 1 × 10^4^ cells per well. At the indicated time points (0, 24, 48 and 72 h after culture), 20 μl of 5 mg/ml MTT (Sigma, St. Louis, USA) was added to each well, and the plates were incubated at 37°C for an additional 4 h. Then, cells were lysed in dimethyl sulfoxide, and the optical density at 490 nm was determined. Each point was determined in triplicate and an average was obtained for analysis. The experiment was independently repeated three times.

### Cell apoptosis analysis

Following culture for 48 h, the cells were harvested, washed with PBS, resuspended in the binding buffer, and incubated for 15 min in the dark with propidium iodide (PI) and Annexin V/fluorescein isothiocyanate (FITC) (Beyotime Institute of Biotechnology, Jiangsu, China). Apoptosis was then detected by a flow cytometer (Beckman Coulter, Brea, CA, USA). The experiment was independently repeated three times.

### Transwell cell migration/invasion assays

The Transwells (Costar, USA) with 8 μm pore polycarbonate membranes (Corning, NY, USA) were left uncoated or were coated with Matrigel (BD Biosciences, Franklin Lakes, NJ, USA) before use in migration assays and invasion assays, respectively. Matrigel was added into each Transwell upper chamber and placed in a 37°C incubator for 2 h to solidify. The cells were seeded in the transwell upper chamber. The transwell assays were performed according to the manufacturer's instructions. After transwell chambers were incubated for 24 hours at 37°C in a humidified incubator with 5% CO_2_, the lower chamber was stained with crystal violet. Cell migration/invasion was evaluated by counting the cells that had migrated/invaded into the filters. Each assay was performed in triplicate and repeated three times.

### Human HCC xenograft tumor models in nude mice

The following two animal experiments were performed to study the therapeutic effect of RNAi targeting IGF-1R on HCC. BALB/c nude mice (weight 26–32 g, 6-weeks old) were purchased from the Guangdong Medical Laboratory Animal Center (Guangzhou, China). A total of 24 male BALB/c nude mice were randomly divided into the IGF-1R siRNA, negative control, and blank control groups (*n* = 8/group). The mice were injected subcutaneously into the right flank with 5 × 10^6^ Huh7 cells stably expressing IGF-1R siRNA, Huh7 cells stably expressing negative control, and untreated Huh7 cells (blank control), respectively. Moreover, we conducted another animal experiment by injecting 5 × 10^6^ Huh7 cells subcutaneously into nude mice. Once tumor volume reached 160-220 mm^3^, the mice were randomized into the IGF-1R siRNA, negative control, and blank control groups (*n* = 4/group), and they were injected intratumorally with about 6 × 10^7^ copies of lentivirus-IGF-1R siRNA, 6 × 10^7^ copies of lentivirus-negative control, and 30 μl PBS, respectively. For the above two animal experiments, the mice were monitored every other day for tumor size. The largest (a) and smallest (b) superficial diameters of tumors were measured with vernier calipers, and the tumor volume (mm^3^) (= a × b^2^ × 0.5) was calculated. All animal experiments were approved by the medical ethics committee of the First Affiliated Hospital of Jinan University, and carried out in accordance with the guidelines for Care and Use of Laboratory Animals of the First Affiliated Hospital of Jinan University.

### Protein array measure

1 × 10^7^ Huh7 cells stably expressing IGF-1R siRNA or negative control were solubilized with 1 × cell lysis buffer. The cell lysates were analyzed with Quantibody^®^ Human Cytokine Antibody Array 440 (RayBiotech Inc., Norcross, GA, USA) according to the manufacturer's instructions. Briefly, the glass slides were blocked for 30 min and then incubated with 100 μl of the standard cytokines or the cell lysates (500 μg/ml of protein) at room temperature for 1–2 hour. Then, the glass slides were washed and 80 μl of the detection antibody cocktail was added to each well, incubating at room temperature for 1–2 hour. Finally, 80 μl of Cy3 equivalent dye-conjugated streptavidin was added and incubated for 1 hour. The signals (wavelength of 532 nm) were scanned using InnoScan 300 Microarray Scanner (Innopsys Inc., Chicago, Illinois, USA).

### Statistical analysis

Categorical data were evaluated by χ^2^ or Fisher's exact tests, depending on the absolute numbers included in the analysis, quantitative data were analyzed by independent sample *t* test followed by Mann-Whitney *U* test. SPSS 16.0 software (SPSS, Inc., Chicago, IL) was used for all statistical analysis. Results were considered statistically significant at *P* < 0.05.
